# Osteoarthritis: Insights into Diagnosis, Pathophysiology, Therapeutic Avenues, and the Potential of Natural Extracts

**DOI:** 10.3390/cimb46050251

**Published:** 2024-04-29

**Authors:** Chiara Coppola, Marco Greco, Anas Munir, Debora Musarò, Stefano Quarta, Marika Massaro, Maria Giulia Lionetto, Michele Maffia

**Affiliations:** 1 Department of Mathematics and Physics “E. De Giorgi”, University of Salento, Via Lecce-Arnesano, 73100 Lecce, Italy; chiara.coppola@unisalento.it (C.C.); anas.munir@studenti.unisalento.it (A.M.); 2 Department of Biological and Environmental Science and Technology, University of Salento, Via Lecce-Monteroni, 73100 Lecce, Italy; marco.greco@unisalento.it (M.G.); debora.musaro@unisalento.it (D.M.); stefanoquarta@cnr.it (S.Q.); giulia.lionetto@unisalento.it (M.G.L.); 3 Institute of Clinical Physiology (IFC), National Research Council (CNR), 73100 Lecce, Italy; marika.massaro@ifc.cnr.it; 4 Department of Experimental Medicine, University of Salento, Via Lecce-Monteroni, 73100 Lecce, Italy

**Keywords:** osteoarthritis, natural extracts, curcumin, bromelain, *Boswellia serrata*, *Harpagophytum procumbens*, devil’s claw, aescin, *Matricaria chamomilla*, *Glycine soja*, *Zingiber*, quercetin

## Abstract

Osteoarthritis (OA) stands as a prevalent and progressively debilitating clinical condition globally, impacting joint structures and leading to their gradual deterioration through inflammatory mechanisms. While both non-modifiable and modifiable factors contribute to its onset, numerous aspects of OA pathophysiology remain elusive despite considerable research strides. Presently, diagnosis heavily relies on clinician expertise and meticulous differential diagnosis to exclude other joint-affecting conditions. Therapeutic approaches for OA predominantly focus on patient education for self-management alongside tailored exercise regimens, often complemented by various pharmacological interventions primarily targeting pain alleviation. However, pharmacological treatments typically exhibit short-term efficacy and local and/or systemic side effects, with prosthetic surgery being the ultimate resolution in severe cases. Thus, exploring the potential integration or substitution of conventional drug therapies with natural compounds and extracts emerges as a promising frontier in enhancing OA management. These alternatives offer improved safety profiles and possess the potential to target specific dysregulated pathways implicated in OA pathogenesis, thereby presenting a holistic approach to address the condition’s complexities.

## 1. Introduction

In recent decades, we have witnessed a continuous improvement in the overall quality of life (QoL) in most developed countries. While this has improved longevity and reduced mortality from infectious diseases, it has also allowed a range of non-infectious, chronic degenerative diseases to emerge, favored by several conditions and environmental factors, including hectic lifestyles, unhealthy nutrition, sedentary habits, and constant exposure to pervasive environmental pollution [1]. Consequently, for most developed countries today, these diseases represent the main cause of mortality and disability.

Prominent clinical conditions impacted by these trends include Parkinson’s disease (PD), osteoarthritis (OA), and type II diabetes mellitus (T2DM). Remarkably, since 1990, their prevalence has surged by 155.5%, 132.2%, and 129.7%, respectively. Presently, the global tally of diagnosed cases includes over 590 million individuals with OA, more than 530 million with T2DM, and exceeding 8.5 million with PD [2,3,4,5]. These conditions, which often occur concurrently in the same individual, present formidable challenges to healthcare systems and significantly compromise the QoL for those affected.

The term OA derives from the Greek words “*ostheo*-”, meaning “*of the bone*”, and “-*arthritis*”, which in turn is a combination of the two words “*arthr*-” and “-*itis*”, which stands, respectively, for “joint” and “inflammation” [6]. Encompassing a heterogeneous group of disorders affecting diarthrodial joints and sharing common biological features and clinical outcomes, OA represents one of the most common, invalidating medical conditions in adults. In the body, articular cartilage acts as a shock absorber for the ends of bones within a joint, thanks to its unique structural composition. It is made up of chondrocytes, which are cells that produce and maintain the turnover of the cartilaginous matrix, and an extracellular matrix (ECM) that is predominantly composed of collagen and proteoglycans [7]. This combination imbues the cartilage with a high-water content, granting it both mechanical resistance and durability. On the other hand, the sparse vascularization of the tissue and chondrocytes’ characteristically low metabolic activity limit the capacity of the cartilage to effectively regenerate after injury or natural degradation that occurs over time [8].

For a long time, OA was a mainly wear and tear process of the joint cartilage, and for this reason, the condition was denoted as “osteoarthrosis”, with the Greek term “-*osis*” indicating a degenerative process without inflammation. However, since the 1980s, it has become apparent that an inflammatory component plays a significant role in the pathogenic process. Consequently, the name of the disease has been revised to reflect this newfound understanding [9].

To date, two main forms of OA have been recognized, namely primary or idiopathic and secondary, as per the causes associated with its onset. While the etiology is complex, the risk factors vary with the forms of the disease. For primary OA, the primary contributors are age, sex, ethnicity, and genetics [10]. According to the World Health Organization, it typically develops between the late 40s and mid-50s, with about 73% of patients over the age of 55, and of these, 60% are female [11]. This can be attributed to natural aging processes, which cause a reduction in synovial fluid, and changes in its composition and quality. Moreover, in advanced age, the body has gone through considerable traumatic stress and wear, and the inflammatory processes that are triggered then lead to alterations in bone and cartilage, causing osteophytes [12]. The higher incidence of OA in females is believed to be primarily linked to hormonal factors. These factors include hormonal fluctuations during menstruation cycles and, notably, postmenopausal changes [13]. Estrogens play a crucial role in cartilage protection, inflammation modulation, and bone metabolism promotion [14,15,16]. Additionally, pregnancy, with its associated weight gain and prolonged hormonal fluctuations, weakens joints, particularly those in the lower part of the body and the spine [17,18]. Sex differences extend to joint alignment, with females generally exhibiting a higher quadriceps angle, lower arch height index, and a broader range of internal and external rotation in the hip joint. These factors result in prolonged stress on the knee and hip joints over time [19].

Secondary forms of OA, on the other hand, may be attributed to causative events capable of weakening the structure of the joint [10]. Among these, traumatic events at the level of articulation, sports activities or demanding jobs, conditions like overweight or obesity, metabolic diseases like diabetes or gout, joint malalignment, congenital deformity, body length, bone inequity, and reduced support to the structure due to weakness of ligaments or surrounding muscle tissue [20,21].

Investigating the pathogenesis of OA remains an ongoing challenge, with much yet to be explored and comprehended. Notably, the factors influencing the diverse timing of interindividual progression, the intricate dynamics of communication among cartilage and surrounding tissues, and the distinct roles played by molecules implicated in various forms of inflammation require further elucidation [22].

As of now, the therapeutic landscape for OA aims at mitigating pain symptoms, enhancing joint function, and, ideally, impeding or delaying their worsening. This typically involves the systemic or local administration of analgesic and/or anti-inflammatory molecules [23]. This conventional approach is often complemented by manual and instrumental physiotherapeutic interventions strategically designed to alleviate pain and fortify the muscles supporting the compromised joint [24]. Surgical prosthetic intervention, albeit considered a last resort, is the only definitive solution, yet it remains fraught with uncertainties concerning outcomes and recovery [25,26].

In recent years, there has been a growing acknowledgment of the significance of natural-type molecules, nutraceuticals, and various plant-based extracts. These substances have indeed shown the potential to act through mechanisms complementary to conventional drugs, offering lower side effects and, perhaps, more specific targeting of pathways fundamental to the pathophysiology of OA [27,28].

This article aims to report some of the most recent findings regarding alternative therapeutic regimes using natural extracts, correlating with the latest understanding of the pathogenesis mechanisms of OA.

## 2. Diagnosis

In the context of OA, pain represents a cardinal symptom, often manifesting as either persistent or intermittent discomfort, prompting people to seek medical attention. It results from inflammation and abnormal friction between the joint surfaces, and it is one of the warning bells that leads the physician to a diagnosis [29]. Individuals with OA may encounter pain while moving the affected joints or even at rest, and the severity varies with the stage of the disease. Additionally, joint stiffness, swelling, reduced flexibility, and, in advanced stages, joint deformity are common manifestations of OA [30]. For physicians, the diagnostic task relies exclusively on clinical assessments, and no laboratory test provides direct support, even if some circulating molecules can represent useful tools for differential diagnosis. In any case, an OA setting, given its inflammatory nature, may be associated with an increase in C-creative protein (CRP) and erythrocyte sedimentation rate (ESR) in serum [31,32]. On the other hand, the evaluation of additional parameters such as complete blood count (CBP), as well as the search for rheumatoid factor (RF), anti-cyclic citrullinated peptide (Anti-CCP), or antinuclear antibody (ANA) allows the exclusion of a rheumatoid arthritis condition [33,34,35,36].

The lack of clear-cut biomarkers underscores the need for a thorough assessment of symptoms, medical history, and physical examinations to achieve a precise diagnosis [37,38]. Furthermore, the need to distinguish OA from other clinical conditions, including inflammatory, infectious, or crystal deposition (e.g., gout) arthritis, as well as soft tissue injuries like bursitis, tendinitis, and meniscal tears, adds complexity to the diagnostic process [39,40,41,42]. Moreover, the uniqueness of the disease manifests in each joint, encompassing distinct onsets, progressions, and physical examination findings.

Numerous biomarkers have emerged in recent decades, with a focus on structural molecules associated with cartilage, bone, or synovium, specific to particular joints or referable to different ones. They are often detectable in circulating blood or urine, enabling noninvasive collection and correlation with cartilage, bone, or ECM-altered metabolism, as well as ongoing inflammatory pathways [43,44,45,46,47].

The Osteoarthritis Biomarkers Network is actively driving the research of novel biomarkers for OA, proposing a classification system based on the burden of disease, investigative, prognostic, efficacy of intervention, and diagnostic (BIPED) categories [48].

Given the chondrodegenerative nature of OA, circulating molecules reflective of cartilage turnover represent extremely promising diagnostic and prognostic indicators. Notably, epitopes of Fibulin-3 (Fib3-1, Fib3-2, and Fib3-3), aggrecanase-generated aggrecan (ARGS), cartilage oligomeric matrix protein (COMP), and C-telopeptide of type II collagen (CTX-II), along with metabolites like acylcarnitines, uric acids, cystine, and tyrosine, frequently exhibit altered levels in this condition [49,50,51,52,53,54,55].

Moreover, markers associated with low inflammation levels, such as TNF-α, IL-6, and IL-1β, are under extensive investigation and are proposed as discriminators between OA and other joint disorders characterized by a severe inflammatory component [56,57]. Additionally, small noncoding RNAs, particularly microRNAs (miRNAs), are emerging as useful tools in correlating their fluctuations with OA severity and in distinguishing them from other joint disorders [58,59,60].

Recently, Kolhe et al. (2017) demonstrated that well-defined pools of miRNAs can serve as sex-specific markers of OA. This is particularly important due to the higher incidence of the condition in women [61].

To date, however, none of these biomarkers has proven to be sufficiently discriminating, neither in the context of routine diagnosis nor in the evaluation of the disease progression.

Imaging serves as valuable assistance for physicians by confirming diagnoses and excluding alternative pathologies. In this regard, plain radiography emerges as a useful tool. While less commonly employed, alternative diagnostic methods like MRI and CT are utilized in the diagnosis of OA, and in many cases, MRI excels in detecting OA at earlier stages compared to conventional radiographs [62]. Additionally, ultrasonography can contribute to the diagnosis by identifying synovial inflammation, effusions, and any osteophyte formations associated with OA [63]. Ultrasound, characterized by its noninvasive, swift, and cost-effective nature, serves as an imaging technique for observing joint changes. However, a limitation lies in its inability to visualize bone conditions, restricting its diagnostic scope to soft tissues [64]. Furthermore, the data it produces may not fully align with the Kellgren and Lawrence (KL) radiological-based scale, established in 1957 by the eponymous physician duo and acknowledged by the World Health Organization (WHO) since 1961 as a classification system for the severity of OA [65,66].

The KL classification system, originally defined to assess conditions related to the knee, is a tool widely used in clinics, epidemiological studies, and research contexts [67,68,69]. After conducting a radiographic analysis, according to its guidelines, it is possible to assign a score ranging from 0 to 4 to the subject, reflecting the severity of OA, as outlined in Table 1 [70].

Despite its widespread adoption and ongoing revisions, the KL classification system has faced some criticism over time. Firstly, it relies on the presence or absence of osteophytes, which may not always be detectable. Furthermore, such formations are not clearly defined and leave considerable leeway for the subjectivity of the examiner. The scale also ignores other factors that affect OA, such as cartilage, synovial fluid, and soft tissue changes, as well as the patient’s symptoms [72,73,74]. Moreover, it is not compatible with other imaging modalities [73]. However, despite many efforts to develop more objective and reliable scales for OA diagnosis and evaluation [70], none of them have found the same widespread acceptance in the clinical and research community as the KL scale.

## 3. Pathogenesis

The axial skeleton represents the framework of the body, preventing the collapse of internal organs and offering protection. The joints, from the Latin “*iuncus*”, meaning “united”, interconnect the bones to allow their movement, also determining their direction and range when muscle tensions or external forces impact them. Of the different types of joints existing in our body, diarthrodial or synovial ones are lubricated by synovial fluid for smooth movement, exhibiting a complex architecture while sharing many fundamental elements.

The articulating ends of the bones are enveloped in a smooth, hyaline cartilage layer designed to minimize friction and offer cushioning, thus giving rise to a synovial cavity encapsulated by a sturdy capsule, serving to stabilize it [75]. At the interface between the bone and the hyaline cartilage, a chondro-osseous junction is present in the form of mineralized cartilage, bound to the bone by a lower cement line and, by the upper tidemark, to the articular soft cartilage [76,77]. The capsule comprises an outer layer of densely packed connective tissue, which is sparsely vascularized but richly innervated, firmly attached around the entire circumference of each bone articular end. On the inner side of the capsule lies the synovial membrane, endowed with secretory capabilities, responsible for producing synovial fluid [78]. Changes in the elements comprising joints are linked to the onset and progression of OA. While these alterations may initially impact just a single component, such as cartilage or bone, the interconnected nature of joint structures results in a condition that affects the entire joint, both physically and functionally [79]. In time, this causes a condition of persistent pain, disability, loss of function, and decreased QoL.

The pathophysiology of OA is still far from being fully understood despite some factors being well known. OA has long been linked primarily to the degeneration of articular cartilage, often stemming from factors such as trauma, overweight, metabolic disorders, and genetic predispositions. However, current understanding suggests that subchondral bone lesions play a pivotal role in the early stages of this disease, contributing to the development of ectopic bone and osteophytes [80,81]. It is important to highlight that the subchondral bone provides mechanical and trophic support to the joint cartilage, so its alteration can significantly impact the metabolic health of the joint cartilage [79].

In this context, the chronic low-grade inflammation of the synovial lining emerges as a central player in the pathophysiology of OA, with immunological mechanisms increasingly recognized as the main drivers in inflammation-induced tissue damage [82]. Moreover, neuroinflammation and central sensitization mechanisms are pivotal in initiating and perpetuating pain in the disease progression [83].

Articular cartilage is a tissue extremely resilient to mechanical stress and capable of withstanding loads far beyond normal body demands. It is composed of a matrix, the key components of which include type II collagen, hyaluronic acid, aggrecan, and various highly hydrated proteoglycans, secreted by a population of specialized cells called chondrocytes, which account for 1% of its total composition [84,85,86,87,88]. Despite its remarkable durability, however, articular cartilage exhibits a limited capacity for self-repair [6,89]. As aforementioned, cartilage lacks a robust blood vessel network, and the chondrocytes, which live in a mostly hypoxic environment, rely mainly on a nutrient diffusion process for sustaining their metabolism and their secretory activity [90,91].

The pericellular matrix, a specialized layer of ECM enveloping one to eight chondrocytes, forms the essential architecture of the chondrons, which represent the functional secreting units of the joint cartilage [92,93]. It preserves chondrocyte activity, shielding these cells from deleterious interactions with ECM components while regulating the flow of nutrients. The pericellular matrix consists of most of the elements of the ECM, with the addition of type VI and IX collagen, which greatly bolsters its resilience against external forces [94,95].

Chondrocytes, through interaction with their envelope via surface integrins acting as mechanical–chemical receptors, perceive mechanical stresses on the cartilage, thereby stimulating the deposition and turnover of matrix components [96]. Integrins play an important role in signal transduction, serving as mediators not only in mechanotransduction processes but also in processes of cell adhesion, migration, and inflammation response.

According to recent findings, a loss in the homeostasis of integrins signaling transduction is associated with OA onset [97,98,99]. During earlier stages of OA, cartilage undergoes several changes in its composition and structure. Physiologically, the structure and the composition of the ECM are handled by metalloproteinases (MMPs), adamalysins (ADAMs), and ADAM with thrombospondin motifs (ADAMTSs), whose activity is tightly regulated and balanced by tissue inhibitors of MMP (TIMPs) [100,101]. Physiologically, indeed, cartilage exists in a state of fluid balance between catabolism and anabolism; pathologically, this equilibrium is moved toward a greater MMP activity and, so, toward its catabolism. In the initial phases of OA, chondrocytes respond by increasing their synthesis of matrix, trying to restore the integrity of the compromised structure. The damage to the pericellular matrix, however, destroys their niche, causing a loss in their capability to efficiently perceive the state of the surrounding area [102,103].

Werb and coworkers (1989), in their pioneering in vitro work, observed that integrins could mediate, as a form of mechanical response, the increase in transcription and secretion of several MMPs, such as MMP-1, MMP-3, MMP-10, and MMP-13, with the latter having a pronounced activity in degrading type II collagen [104,105]. At the end of 1980, moreover, several studies found how the homeostasis of the cartilage was preserved thanks to the activity of the transforming growth factor beta (TGF-β), having a role in inhibiting the activity of the TIMPs [106].

TGF-β has also been shown to modulate the expression of integrins. The molecule has an important role in chondrocyte differentiation and maturation, as well as promoting cartilage synthesis. However, when present at high levels, it can have detrimental effects on cartilage integrity, compromising chondrocyte metabolic activity. Integrins themselves can respond to mechanical stimulation by inducing the release of TGF-β, but when this pathway is misruled, they contribute to ECM destruction [22,107].

One of the most deleterious consequences of the dysregulated activity of MMPs is the sustained activation of the chondrocyte pathway downstream of the α5β1 integrin, also known as the fibronectin receptor. This interaction occurs with a soluble fibronectin fragment generated from the proteolytic cleavage of the full-length protein [108,109]. Subsequently, this cascade triggers a significant activation of PKCδ, which in turn induces the activation of nuclear factor kappa B (NF-κB) and mitogen-activated protein kinase (MAPK), eliciting a complex cellular response [110,111,112]. The NF-κB pathway stands as a potent proinflammatory agent, exerting significant influence in the initiation of OA. Its activation can also be triggered by various proinflammatory cytokines, such as IL-1β, TNF-α, and IL-6, whose concentrations increase within the joint during inflammation, but it also perpetuates its activity through a positive feedback loop, driving chondrocytes to enhance their synthesis [113,114]. This cascade of events underscores the critical role of NF-κB in the perpetuation and exacerbation of OA pathology [115]. On the other hand, MAPK can suppress the synthesis of ECM components while simultaneously stimulating the synthesis and release of MMPs [116,117]. Together, the NF-κB and MAPK signaling pathways contribute to ECM degeneration and the progression of OA by generating reactive oxygen and nitrogen species (RONS), prostaglandin E2, MMPs, and ADAMTSs [118,119,120,121].

**Figure 1:**
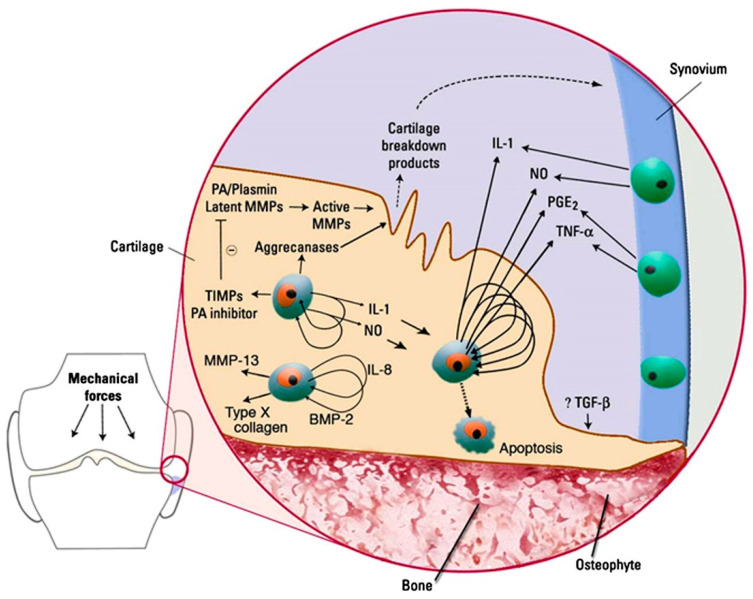
Schematic representation of the pathological mechanisms in osteoarthritis (OA) highlighting the complex interplay among bone, cartilage, and synovial tissues [126]. The diagram illustrates how prolonged mechanical stress, wear, and trauma lead to the secretion of extracellular matrix (ECM) by cartilage cells. Key enzymes involved in ECM remodeling—such as MMPs, ADAMTSs, plasmin, plasminogen activator, and TIMPs—are shown to have disrupted activity, contributing to excessive cartilage catabolism. The role of interleukins, TGF-β, and BMP-2 in cartilage morphology, tissue homeostasis, and metabolism is depicted alongside the suppression of TIMPs. The feedback loop of MMP activation, TNF-α, and interleukin synthesis by synovial tissues and chondrocytes fosters a sustained inflammatory cascade. This leads to the release of NO and prostaglandin E2, proliferation of chondrocytes, and deposition of collagen type X, which undergoes calcification, exacerbating OA pathology. BMP-2: bone morphogenetic protein 2; PA: plasminogen activator; MMP: matrix metalloproteinase; TGF-β: transforming growth factor beta; TIMP: tissue inhibitor of metalloproteinase; TNF-α: tumor necrosis factor alpha; NO: nitric oxide; PGE2: Prostaglandin E2; IL-1: Interleukin-1; IL-8: Interleukin-8.

The gradual degradation experienced by cartilage initiates a calcification process, affecting both its superficial and deeper layers, eventually leading to the delamination and exposure of underlying bone over time [122]. This progression appears to be facilitated by the deposition of calcium crystals within chondrocytes and their surrounding regions, facilitated by the release of vesicles containing mineral salts. The discovery that these vesicles are enriched with microRNAs hints at a greater complexity underlying the process than previously understood [123]. From the bone marrow, new vessels and sensitive nervous terminations start to grow through the new fissures in the osteochondral junction, surrounded by novel bone [76]. This phenomenon also arises from the migration and excessive proliferation of new chondrocytes, which commence depositing new layers of type X collagen. However, unlike the typical physiological chondrogenic process, these layers are not replaced by type II collagen but instead undergo calcification (Figure 1) [124,125].

Recent findings have shed light on one of the mechanisms that could correlate hormone activity on the cartilage with the greater incidence of OA in the female sex. Wang and colleagues (2021) have observed a role for estrogen receptor α (ERα) in chondrocyte senescence, where the phenotype of these cells is influenced by the levels of this receptor. Utilizing RNA sequencing techniques, they observed differential transcription of the estrogen receptor-1 (ESR1) gene, encoding ERα, in chondrocytes between healthy individuals and OA patients [127]. This finding complements the observation of low ERα levels measured in OA-affected joints [128]. Furthermore, their study revealed that restoring ERα levels in chondrocytes to physiological levels reduces senescence, thereby ameliorating the pathological phenotype. Conversely, knockout of ERα exacerbates senescence, compromising the chondrocyte’s ability to efficiently respond to mechanical stress [127].

## 4. Treatment

While there is currently no cure for OA, treatment primarily focuses on mitigating modifiable risk factors before its onset and alleviating its symptoms once it occurs.

Proactive measures, such as weight loss or modification of conditions of postural or orthopedic abnormalities, stand out as an effective means to diminish the likelihood of developing OA in the lower limbs and spine joints [129,130,131]. Weight loss also represents the first line of treatment once OA manifests, especially in conditions affecting the knee [131]. However, during the acute pain phases of OA, patients often require a pharmacological approach to enhance their QoL. Effectively managing symptoms becomes pivotal, marking a shift toward targeted interventions aimed at minimizing discomfort and optimizing overall well-being.

Managing patients with OA demands a comprehensive approach, considering the progressive nature of the condition and its profound impact on patients’ QoL. While physiotherapy and rehabilitation are valuable components, they may not always suffice in addressing the debilitating pain associated with OA. Therefore, integrating pharmacological interventions such as nonsteroidal anti-inflammatory drugs (NSAIDs) and analgesics becomes essential [132,133,134]. Additionally, personalized treatment plans, overseen by medical specialists, may incorporate intra-articular therapies like steroids, platelet-rich plasma (PRP), and hyaluronates to augment symptom management [135,136,137].

Moreover, since the nature of OA-related pain is persistent and potentially distressing, it is essential to address its psychological impact. Collaborating with healthcare professionals like psychologists can provide valuable support alongside physicians and physical therapists, helping patients cope with pain and any associated depressive symptoms [138,139].

Ultimately, when conservative measures fail to provide adequate relief and functional improvement, surgical interventions may become necessary to address the specific joint pathology effectively.

### 4.1. Physical Treatments in OA

According to the latest and most authoritative guidelines, physical exercise stands out as the primary intervention in managing overt cases of OA, alongside education for self-management and physiotherapy [140,141]. Nevertheless, it is crucial to contextualize the therapy, considering that it may vary significantly depending on the specific joint affected by the degenerative process. Comorbidities also have to be considered to suggest to the individual not only the safest and most appropriate exercise but also the duration and intensity of the training sessions.

Physical exercise has demonstrated greater effectiveness in managing OA affecting the knee or hip compared to the hand [142]. There is no “one-size-fits-all” approach to exercise, as different activities suit different individuals based on their preferences, fitness levels, and health conditions. However, some popular forms of exercise, such as walking, using treadmills, or cycling, are noteworthy for their popular adoption and their positive reception by patients. Additionally, aquatic exercises serve as a valuable tool in OA management, strengthening the benefits of aerobic activity while significantly reducing the strain on the joints in a water-based environment [143]. Tai chi is highly recommended in individuals with knee or hip OA, as it has demonstrated effects on both physical and mental well-being [142]. Similarly, while yoga appears to offer significant benefits to patients, there is a dearth of literature recommending its use [142,144,145].

Finally, numerous studies have highlighted the utility, particularly in cases of knee OA, of strengthening the muscles that support the affected joint through targeted rehabilitation or training programs. Weakness in muscles such as the femoral quadriceps can lead to increased knee joint loading. However, the optimal approach to achieve this goal remains a subject of debate among medical societies [146].

### 4.2. Pharmacological Treatments in OA

#### 4.2.1. Nonsteroidal Anti-Inflammatory Drugs

In the context of OA, the second-line treatment involves NSAIDs and analgesics. These compounds act by inhibiting prostaglandin-endoperoxide synthase, also known as cyclooxygenases (COXs), oxidoreductases facilitating the conversion of arachidonic acid into prostanoids [147]. Among the various isoforms of COXs, the inducible expression of COX-2 is closely associated with inflammatory cytokines such as IL-1b and TNF-a, in addition to oxidative stress [148]. Consistent with these findings, several studies have documented an elevated level of these inflammatory mediators, coupled with an increased amount of proinflammatory nitric oxide (NO) and a significant rise in the level of COX-2 within the cartilage of individuals affected by OA [149,150]. These observations underscore the interconnection between inflammatory processes, oxidative stress, and clinical conditions.

However, the use of NSAIDs comes with several drawbacks associated with the inhibition of prostaglandin synthesis. Non-selective COX inhibitors impact both COX-1 and COX-2 isoforms, affecting the gastric mucosa. Notably, COX-1 is crucial for protecting the stomach against ulcerations and bleeding [151]. Conversely, COX-2-specific inhibitors, designed to spare isoform 1 activity, are linked to an elevated risk of thrombosis [152].

In the body, while both COX-1 and COX-2 contribute to prostaglandin synthesis, COX-2 represents the primary source of prostacyclins, playing roles in inflammation and vasodilation [153,154]. Meanwhile, COX-1 catalyzes the synthesis of thromboxanes, exerting a potent vasoconstrictive effect [155]. The inhibition of COX-2 therefore shifts the balance toward increased vasoconstriction and prothrombotic activity [156].

Opting for NSAIDs in cream or gel formulation stands out as a preferred approach for managing OA, especially among the elderly. While these topicals may exhibit a slower absorption rate, their pharmacological activity is comparable to oral alternatives [157,158]. Their use is recommended by the main international guidelines for knee and hand OA treatment according to superior safety profile. The American College of Rheumatology strongly endorses the topical application of NSAIDs over oral consumption, particularly emphasizing this for individuals aged 75 years or older with knee OA. This recommendation holds particular significance for those with coexisting conditions and heightened risks of cardiovascular, gastrointestinal, or renal side effects, which are commonly observed in this age group [159].

#### 4.2.2. Steroidal Anti-Inflammatory Drugs

Among the most powerful molecules capable of mitigating inflammatory responses in the human body are corticosteroids. Derived from cortisones, this class of drugs exerts its influence by downregulating the expression of numerous genes through interactions with their transcription factors [160,161]. However, prolonged use of corticosteroids is associated with significant side effects, including weight gain, swelling, hypertension, diabetes, and increased susceptibility to infections [162,163,164,165]. The literature contains poor data about the enteral use of such a class of molecules in OA contexts. The limited number of trials existing has shown to provide just slight benefits in pain relief in both hand and knee OA, although limited in time [166,167].

Another therapeutic strategy involves the direct administration of corticosteroid injections into the affected area. Initially, the pioneering work of Hollander and colleagues in 1951 was performed in knee arthritis rheumatoid [168], with Miller and coworkers replicating the treatment for OA subjects seven years later [169].

Since then, other joints affected by OA have also become sites of injection, and this form of therapy remains an area of active research [170,171,172,173]. These investigations aim to assess the duration of the anti-inflammatory and analgesic effects of corticosteroids, considering that the latest evidence indicates a relatively short duration, from a few weeks to months, necessitating careful consideration when determining the appropriate posology for treatment [166].

If a local use of corticosteroid keeps the drug effects mostly localized to the joint, reducing at the same time systemic side effects, some drawbacks in the procedure remain. Despite now being quite rare, infections can follow the procedure; in addition, some studies have associated the treatment with local side effects like necrosis, tendon weakening, and cartilage reduction [174,175,176,177]. On the other hand, despite being rare, some systemic responses to the drug may be observed, like headache, insomnia, short-term glucose levels increase, hypothalamic–pituitary–adrenal axis suppression, and iatrogenic Cushing syndrome [178,179,180].

According to Osteoarthritis Research Society International (OARSI), European League Against Rheumatism (EULAR), and Royal Australian College of General Practitioners (RACGP) guidelines, intra-articular corticosteroid use, together with physical exercise, is recommended for short-term pain reduction in knee OA, conditionally and limitedly in time [181], while other societies maintain a more cautious approach to the topic, like the American Academy of Orthopedic Surgeons (AAOS) [182].

#### 4.2.3. Disease-Modifying OA Drugs

In recent years, with the attempt to relieve patients’ pain and block the progression of OA, efforts have been directed toward the research of innovative pharmacological strategies. Although the use of NSAIDs and corticosteroids provides some symptomatic relief, their benefits are modest, limited in time, and carry the risk of adverse events (AEs).

Several potential molecular targets have been identified thanks to the comprehension of the pathways involved in the condition’s onset and progression. These include matrix-degrading proteases, mechanisms of altered senescence of chondrocytes, cartilage repair mechanisms, bone remodeling processes, and low-grade inflammation mediators. Disease-modifying OA drugs (DMOADs) are a group of molecules capable of intervening in specific molecular mediators of these processes. Among the most promising targets are matrix metalloproteinases such as MMP-13 protease or ADAMTS-4 and -5 peptidase, growth factors like fibroblast growth factor-18 (FGF-18), bone morphogenetic protein (BMP-7), or TGF-β, cytokines, and small molecule like TNF-α or IL-1β [183,184,185,186,187,188,189,190,191,192].

Additional approaches to preserve cartilage health include targeting cellular senescence, a mechanism associated with stress response [193]. In OA, oxidative stress is associated with a premature joint cellular component aging process, affecting not only chondrocytes but also synovium fibroblasts, osteoblasts, osteoclasts, and musculoskeletal cells [194,195,196,197]. This gives origin to a senescence-associated secretory phenotype (SASP), resulting in a massive release of proinflammatory cytokines and proteases in the joint space [198].

Current research is focused on pursuing a pharmacological approach to the phenomenon by developing molecules with senolytic and/or senomorphic activity. Senolytic molecules can suppress anti-apoptotic or pro-senescence pathways, often upregulated in pathology. Among their targets are the anti-apoptotic PI3K/Akt pathway and B cell lymphoma family proteins Bcl-2, Bcl-XL, and Bcl-W, as well as pro-senescence proteins p15, p16, p21, and p53 [199,200,201,202,203,204]. On the other hand, senomorphics act by inhibiting cells’ SASP or neutralizing their biological effect; promising targets include AMPK signaling, IL-6 receptors, IL-8, and IL-1β, which in turn inhibit MMP-13 and ADAMTS5 production. Preliminary studies on senomorphic preparations capable of directly inhibiting matrix-degrading enzymes are also ongoing [205,206,207,208,209,210].

Finally, OA presents itself as an extremely painful condition. Despite the reduced innervation of the cartilage, the joint is rich in sensorial terminations, which, during the pathological degeneration of the tissue, start to perceive the effects of cytokines and proinflammatory stimuli [211,212]. The peripheral sensitization is followed by a central sensitization with the establishment of conditions of allodynia or hyperalgesia due to mechanical stresses. Increased levels of nerve growth factor (NGF) are commonly observed in synovial fluid, which is released by joint cells during OA progression and associated with peripheral nociceptor hyperactivation [213,214]. Consequently, targeting NGF is therefore nowadays considered a promising target to modulate OA patients’ perceived pain, with several trials aiming to inhibit its deleterious effects during joint degeneration [215,216,217].

Although DMOADs have undoubted theoretical application potential, their clinical use in the context of OA still seems far off. This is due to major challenges in development, including regulatory guidelines, current assessment by conventional radiography, and lack of patient stratification in clinical trials. It is indeed complex to translate data obtained from animal trials with artificially induced pathology to human models. Furthermore, both American and European pharmaceutical regulatory authorities require a demonstrated reduction in pain symptoms and joint space thinning for the approval of a drug for OA [218]. However, radiographic measurements used to assess this parameter can be challenging to standardize and evaluate in large trials [219]. In addition, there is no direct correlation between joint morphological changes and OA progression or severity. The need for biomarkers to assess the progress of trials more objectively on DMOADs is therefore deeply felt.

### 4.3. Regenerative Therapies in OA

Regenerative therapies are gaining increasing popularity in the field of orthopedic medicine for the treatment of OA and joint pathologies in general. Among these, some promising options are viscosupplementation with HA and Platelet-Rich Plasma (PRP) therapies. These therapies provide innovative and less-invasive solutions to enhance joint functionality and alleviate pain, enabling patients to regain a better QoL.

#### 4.3.1. Hyaluronic Acid

In OA, a relatively recent therapeutic approach is represented by HA use as viscosupplementation, either through injection or enteral supplementation. HA is a high-molecular-weight (MW) molecule (6.5 kDA to 20 MDa) naturally synthesized by the body, composed of alternately repeating _D_-glucuronic acid and *N*-acetylglucosamine units [220]. It is physiologically present in soft connective tissue, cartilage, and synovial fluid, where it serves as a lubricant and antioxidant [221]. Additionally, HA plays a crucial role as a modulator of cytokine release, influencing cell proliferation and migration and reducing MMP activity [222].

In the context of OA, the onset and progression are associated with the alteration of joint HA and its degradation through depolymerization. This process leads to the formation of lower MW forms of HA, causing changes in the composition and mechanical properties of synovial fluid, as well as a reduction in cartilage renewal. Such processes are mainly related to increased hyaluronidase activity and RONS levels [223,224]. Moreover, while high-MW HA has a protective effect on the join, low-MW forms of the molecule appear to be proinflammatory [225,226].

The exogenous supply of HA results is unable to completely overlap the loss of the endogenous one but is still able to partially restore the functionality of the synovial fluid, stimulating matrix production and promoting an anti-inflammatory effect through NF-κB and MAPK signaling pathways inhibition by reducing TNFα, IL-1β, and IL-6 levels in the joint [227,228]. Moreover, being TNFα a stimulator of NO and metalloproteinase synthesis, its reduction further preserves the joint from OA-mediated degradation [229].

The Food and Drug Administration already approved the intra-articular use of HA for the therapy of knee OA in 2001, and several applications have been developed since then [230]. Currently, the treatment algorithm recommended by the European Society for Clinical and Economic Aspects of Osteoporosis and Osteoarthritis (ESCEO) advocates the use of intra-articular HA for managing knee OA in patients who continue to experience pain despite the use of NSAIDs. Indeed, given the lack of unequivocal and objectively comparative data regarding the efficacy of corticosteroids and HA injections in relieving OA pain, the latter option offers a superior safety profile [181]. Indeed, given the lack of unequivocal and objectively comparative data regarding the efficacy of corticosteroids and HA injections in relieving OA pain, the latter option offers a superior safety profile [181].

International and national societies guidelines, however, vary, with some, like the EULAR, suggesting the consideration of HA, emphasizing its potentially longer-lasting effects, while others recommend its use just in the second instance, when other therapeutical approaches have proven to be ineffective, like ESCEO or American College of Rheumatology (ACR) [182].

#### 4.3.2. Platelet-Rich Plasma

PRP is a concentrated autologous mixture of platelets, growth factors, and bioactive components. It is obtained through the centrifugation of whole blood and is subsequently reinjected into the same donor, aiming to exploit the regenerative properties of platelets for therapeutic benefits [231]. Once activated by thrombin or collagen, it is capable of releasing molecules, such as cytokines and growth factors like IL-1b and TNF-α capable of reducing inflammatory responses, inhibiting the NF-κB pathway while also promoting mesenchymal stem cell proliferation and matrix deposition and inhibiting metalloproteinase activity, stimulating tissue healing [232,233,234]. PRP has also been shown in both in vivo and in vitro studies to promote, dose-dependently, chondrocyte proliferation and degradation of the damaged ones by stimulating autophagy, with a cascade effect on the increased synthesis of proteoglycan and collagen type II [235].

In 2020, Belk and coworkers published a meta-analysis spanning 18 studies comparing the benefits of PRP intra-articular injection with those provided by HA use. According to their observation, the use of PRP provides results comparable or even superior to those provided by HA treatments [236]. Also, Chen and colleagues, more recently, published a meta-analysis comparing the results of different literature productions involving more than 2700 patients divided into PRP and HA groups [237]. Its result showed that reported Western Ontario and McMaster Universities Arthritis Index (WOMAC) pain marks were higher for PRP-treated subjects, with comparable safety profiles. It is intriguing to consider that recent evidence about the combined use of PRP and HA injection seems promising in knee OA, with functional improvements and pain reduction still after 12 months from the treatment [238].

Due to the high amount of existing PRP formulations and uncertainties about their pharmacodynamics, these treatments have a high grade of heterogeneity. This makes it quite challenging to compare clinical trial results. For these reasons, the main international guidelines discourage the clinical use of PRP [182].

### 4.4. Surgical Approaches

In advanced stages of OA, the depletion of cartilage, joint space, and synovial fluid reaches critical levels, resulting in debilitating pain, functional impairment, and disability. Conservative measures, therefore, become inadequate to address the severity of symptoms, necessitating more invasive interventions, such as surgery.

One such intervention is arthroplasty, a procedure designed to partially or entirely replace, remodel, or realign the compromised joint. This approach, and in particular, total joint arthroplasty, is highly effective and safe for treating knee and hip OA and is also being used for spine, shoulder, ankle, and elbow joints [239]. Surgery is less commonly employed for conditions affecting the hands, where the side effects outweigh the benefits. In such cases, joint protection and splinting offer temporary relief and may postpone the need for surgical intervention [240,241].

Another surgical approach to address joint issues involves corrective osteotomy or arthrodesis. Corrective osteotomy entails reshaping the bones forming the joint to rectify deformities and redistribute the load on less damaged areas of cartilage [242,243,244]. On the other hand, arthrodesis involves immobilizing a mobile or semimobile joint by aligning the component bones and securing them with screws, plates, or bone grafts [245,246]. Osteotomy can alleviate pain and postpone the need for other surgical interventions, offering short-term relief. It may be considered in cases of early joint malalignment without significant cartilage damage or limited joint destruction [239]. It may be considered in cases of early joint malalignment without significant cartilage damage or limited joint destruction [239]. Arthrodesis, being a more radical type of approach, is generally considered when alternative surgical options are impractical or ineffective, particularly in the presence of severe osteoarticular damage [247].

A less-invasive surgical option for treating OA is arthroscopy, a procedure involving the insertion of surgical tools and a micro-camera into the joint capsule. While arthroscopy is commonly used for joints such as the spine, elbow, wrist, and hand, it also extends to larger joints like the ankle and hip. This approach is regarded as conservative in OA treatment, aiming to delay or prevent the need for more invasive procedures while preserving cartilage; for this reason, it is particularly favored in athletic or younger individuals [248].

In cases of spine OA, avoiding fusion surgeries is crucial to mitigate well-known side effects related to its loss of flexibility [249,250]. For elbow OA, treatment options may include joint surface replacement or removal of osteophytes [251]. Conversely, managing wrist and hand OA may involve denervation to alleviate symptoms rather than direct therapeutic intervention [252]. In larger joints, arthroscopy may facilitate the realignment of bone structures or correction of unilateral deformities [253,254]. Although these approaches vary widely, their collective goal is to alleviate pain and enhance joint function in OA patients.

Arthroscopic procedures can involve flushing the joint with saline solution to remove loose particles like cartilage or tissue fibers from the joint fluid, thereby reducing inflammation. Alternatively, a debridement approach can be employed to smooth rough cartilage surfaces and eliminate loose cartilage fragments [255].

Regardless of their invasiveness, surgical procedures inherently carry risks, which tend to increase with the complexity of the intervention. Even though joint replacements have been performed since the 1960s, the procedure cannot be considered entirely safe [256,257]. In the United States alone, it is projected that joint replacement surgeries will reach 3 million by 2030. The most common type of joint replacement procedures, namely knee and hip, have a mortality rate of 1%, with almost 5% of patients experiencing post-surgery complications [258]. Even less-invasive arthroscopic procedures present some side effects, like nerve damage, infections, and clot formation [259,260,261,262,263].

Additionally, implants typically have a lifespan estimated at 15–25 years and necessitate replacement after this period, a concern particularly meaningful for younger patients [264].

Finally, two potential issues may emerge post-surgery. The first is periprosthetic osteolysis, arising from an inflammatory response to wear debris generated by friction between prosthetic components. This reaction activates osteoclasts, leading to periprosthetic bone loss [264,265]. The second potential problem is aseptic loosening, characterized by the loss of implant stability due to disruption of the bond between bone and prosthesis or between prosthesis and cement [266,267]. Aseptic loosening, together with infection, now represents the first cause of short-term failure of early rejection of total joint arthroplasty [268,269,270]. Then, if the prosthesis remains stable and functional, the surgical intervention is typically confined to revising the bearing surfaces and potentially incorporating bone grafting. Alternatively, replacing the entire prosthesis may be considered. Following corrective revision surgery to address the underlying cause of wear, osteolytic areas have the potential to regress and re-ossify [271].

Presently, strategies to mitigate complications in prosthetic components involve developing durable, wear-resistant materials that generate smaller debris, while numerous efforts are constantly directed at optimizing component design and positioning aids to reduce mechanical stresses and micro-movements [272,273]. Furthermore, post-surgical administration of anti-inflammatory or anti-osteoclastic medications like bisphosphonates can inhibit the inflammatory response and bone resorption [274,275].

In perspective, an emerging approach alternative to joint replacement surgery could be represented by the implantation of engineered tissue scaffolds designed to facilitate osteochondral regeneration [276,277]. Recently, an international consortium of research centers and health care providers reported preliminary evaluations demonstrating promising interactions between some of their crafted structures and the surrounding matrix, as well as their encouraging potential in promoting cartilage and bone tissue regeneration [278]. Nevertheless, the durability and safety profile of these artificial structures over extended periods will require further rigorous evaluations before considering human applications.

## 5. Natural Extracts

While NSAIDs remain the front-line therapy for OA management, their safety profile is hardly ideal, based on results from multiple randomized controlled trials (RCTs) and meta-analyses over the years [279,280,281,282]. Briefly, the safety profile of each NSAID varies with its pharmacologic action and individual responses to the drug. The AEs associated with long-term NSAID therapy are gastrointestinal-like lesions and ulcers, cardiovascular-like stroke, myocardial infarction, renal-like edema, and acute kidney disease [283,284,285]. As most of the at-risk population of OA is aging adults and often presents with comorbidities, the risk of AEs increases, and therefore, long-term NSAID regimes become difficult to manage [133].

Nevertheless, OA therapy is essentially the management of pain as the condition itself is irreversible. For this reason, while the use of NSAIDs is inevitable, the pitfalls associated with them could be addressed using bioactive compounds extracted from medicinal plants, herbs, and food sources, collectively termed nutraceuticals [286]. Nutraceuticals are generally considered to be safe because they are obtained from common food sources and represent incredible promise owing to their potent anti-inflammatory and antioxidant properties [287]. A recent Australian study found that 35% of OA patients used nutritional supplements concurrently with conventional analgesics [288]. Aghamohammadi et al. (2020) ascertained that the use of nutraceuticals improved pain scores and consequently led to better physical function in OA patients [289]. Polyphenols are particularly implicated as one of the most important bioactive compounds because of their ROS-scavenging ability and the suppression of proinflammatory pathways, such as MAPK and NF-κB [290,291,292]. These properties have been tested in both in vitro and in vivo OA disease models with a suitable degree of success [293,294,295,296].

Unsurprisingly, therefore, these points make a suitable case for the use of nutraceuticals and plant extracts as an alternative therapy to traditional pharmaceutical agents in OA. A summary of their effectiveness in clinical trials is presented in Table 2.

This section will discuss plant extracts of particular importance and their anti-inflammatory roles from the perspective of OA management, including curcumin from turmeric, bromelain extracted from the pineapple plant, boswellic acid extracted from the Indian frankincense tree, devil’s claw and aescin from horse chestnut, *Matricaria chamomilla*, *Glycine soja*, *Zingiber*, and quercetin.

### 5.1. Curcumin

Being a hydrophobic polyphenol, curcumin, or diferuloylmethane, bears many virtues with other plant-derived polyphenols, particularly being antibacterial, antioxidant, and anti-inflammatory [301]. Derived from the plant *Curcuma longa*, curcumin in the form of powdered turmeric is an essential part of Asian cuisine as a spice and has been valued in traditional Ayurvedic medicine as a remedy to dress wounds and burns and even as an eye ointment [302]. Curcumin has also been tested for its anti-cancer potential in cervical, breast, lung, and pancreatic cancer [303,304,305,306].

In the context of OA, curcumin exerts its action through various mechanisms, as evidenced by multiple studies over the years [307,308,309]. The storm of secretion of inflammatory mediators in OA causes joint destruction, and hence, this represents an important therapeutic target. While curcumin on its own cannot induce apoptosis of synovial fibroblasts within safe doses, Shakibaei and colleagues (2005) found that it can protect chondrocytes from IL-1β-mediated alterations in vitro, hence indirectly exerting its anti-apoptotic effects [310,311]. Curcumin has an established ROS-scavenging activity through its excellent electron-transfer capability; however, its ability to independently perform this in OA models has not yet been reliably demonstrated [312,313]. Chen et al. (2023) showed that a combination of catalase and curcumin prevented oxidative stress by inducing the expression of ROS-scavenging enzymes through the NRF2/HO-1 signaling pathway [314]. Several studies have also explored curcumin-loaded nanoparticles to circumvent the issue of cytotoxicity, and in such a study, Crivelli and colleagues used silk fibroin nanoparticles to encapsulate curcumin and celecoxib to improve the ROS-scavenging ability of curcumin in an in vitro OA model [315]. By inhibiting matrix MMPs, which are involved in the degradation of the extracellular matrix, curcumin also has an anti-catabolic and chondroprotective role [308]. Indeed, Zhang et al. (2016) used curcumin and curcumin-encapsulated nanoparticles, which reduced the RNA expression of MMP 1, 3, and 13, IL-1β, and TNF-α while promoting the expression of CREB-binding protein (CBP)/p300-interacting transactivator with glutamic acid and aspartic acid-tail 2 (Cited2) in human primary chondrocytes [316].

Curcumin’s chondroprotective potential has also been demonstrated in vivo, particularly in mouse models, where it has been shown to decrease disease progression [316]. A 2018 study ascertained curcumin’s therapeutic effect in osteoarthritis to be a result of enhanced autophagy through the Akt/mTOR pathway [317]. Despite its many benefits, curcumin suffers from a major pitfall of poor absorption and solubility, which limits its clinical use. Perhaps this is the reason why only a few RCTs have been conducted over the years to test curcumin formulations in human subjects. A systematic review of RCTs using curcumin for osteoarthritis, conducted by Bannuru and colleagues (2018), revealed that the limited number of such trials was further compromised by their generally low study quality [318]. In such an RCT, it was ascertained that curcumin supplement was significantly better than placebo in improving pain scores for a period of three months in a cohort of OA patients [319]. However, in a comparative evaluation with ibuprofen, while curcumin was shown to have a better gastrointestinal tolerance, the pain scores were similar [297]. A novel encapsulation method used by Yabas and colleagues in their 2021 study claims to improve disease prognosis in a mouse model, addressing the issue of bioavailability [320].

### 5.2. Bromelain

Unlike curcumin, bromelain is not a polyphenol but a proteolytic enzyme obtained as an aqueous extract from the pineapple plant *Ananas comosus* [321]. However, like polyphenols, plant proteases also have beneficial antioxidant, anti-microbial, anti-hypertensive, and ACE-inhibitory properties [322,323,324,325]. Bromelain, which is perhaps the most clinically and industrially important protease, also has an array of additional attributes, like fibrinolytic, analgesic, and immunomodulatory activities [326,327,328].

The enzyme was first used as an anti-inflammatory compound in 1964, directed against patients with rheumatoid and osteoarthritis [329]. In a 2006 pilot study, bromelain on its own was determined to be inefficacious in improving WOMAC scores in moderate to severe OA [330]. When compared with an NSAID like diclofenac, a similar result was achieved by Kasemsuk et al. [331]. Later clinical studies incorporated other extracts like curcumin and *Harpagophytum porocumbens* (HP) with bromelain with suitable pain scores [332]. Italiano and colleagues (2019) assessed the QoL in osteoarthritis patients to find that food supplements containing bromelain and Boswellia serrata improved QoL scores [333]. However, in another clinical trial that combined bromelain with enzymes like trypsin and rutoside trihydrate, the researchers found no significant difference in the pain scores when compared with diclofenac [334].

The anti-inflammatory actions of bromelain from the context of osteoarthritis were explained by a 2021 study, in which the enzyme extract is shown to suppress glycosaminoglycan (GAG) and hence counteract the degradative effects of IL-1β and MMPs [335]. Brochard et al. (2021) demonstrated that bromelain has a similar effect on lipopolysaccharide-induced inflammation, albeit in a combinatorial form with curcumin and *Boswellia serrata* [336]. Similar effects were observed by Quarta and colleagues (2022) in an in vitro model of inflammation [337]. These mixed results suggest that further studies are needed to ascertain the mechanisms of action of bromelain, particularly on its conditions of use.

### 5.3. Boswellia serrata

*Boswellia serrata* is a tree that grows in arid, mountainous regions of India, North Africa, and the Middle East and yields a rubbery oleoresin from its bark, which is called Boswellia Gum Resin Extract (BSE) [338]. For simplicity, in this review, the names of the tree and the extract are used interchangeably. The Boswellia extract is chemically composed of terpenes and the associated terpene acids, namely β-boswellic acid, acetyl-β-boswellic acid, 3-O-acetyl-11-keto-β-boswellic acid, and acetyl-11-keto-β-boswellic acid [339].

Boswellia extract has shown immense potential, primarily due to its anti-inflammatory role, in furthering alternative therapy for diseases like inflammatory bowel disease, asthma, peritumoral brain edema, and osteoarthritis [340,341,342,343]. Kimmatkar and colleagues, in their 2003 RCT, found that when compared with placebo controls, Boswellia extract was significant in decreasing knee pain and swelling [344]. This was also reflected in later studies, either in novel formulations or in combination with other extracts [345,346,347,348]. Neither of these studies, however, compared the efficacy of the extract with any common NSAID; hence, the results cannot be considered conclusive.

Kulkarni et al. showed that Boswellia extract encapsulated in solid lipid nanoparticles reduced proinflammatory cytokines when compared with a non-encapsulated extract [349]. The researchers also found that even though Boswellia extract did not significantly improve pain scores, subjects did not need to resort to a common NSAID during the trial. Similar conclusions were drawn by Henrotin and colleagues in a separate study, specifically from the context of hand osteoarthritis, where NSAID use decreased by 64% over a period of three months [350].

Besides clinical trials, several in vitro and in vivo studies suggest that Boswellia extracts, and more specifically, boswellic acids, inhibit cytokines and enzymes associated with inflammation like COX-1 and Cathepsin-G (catG) [351,352,353]. Even though the concentrations of the bioactive components in Boswellia extract necessary to perform the inhibitory actions have been found to be sub-par, a prevailing theory is that their accumulation might be in lipophilic extra- or intracellular components [339,354].

### 5.4. Harpagophytum procumbens

The complex extract from HP, or devil’s claw, contains phenolic acids and glycosides, triterpenes, phytosterols, iridoid glucosides like harpagoside, and various flavonoids. Various in vitro studies have testified to the anti-inflammatory and analgesic characteristics of the components of this extract by counteracting the production of cytokines [355,356,357]. Of note is a 2017 study, which evidenced that the major active component of HP extract, namely harpagoside, suppresses IL-6 production in an in vitro model of OA chondrocytes [358]. The same group had earlier postulated that the anti-inflammatory activity of harpagoside is due to its inhibition of the NF-κB pathway [359]. Mariano and colleagues, however, argued that the chondroprotection in in vitro models is due to all the various individual components in the HP extract [360].

More recently, Farpour et al. studied the differences in WOMAC and Visual Analog Scale (VAS) scores of cohorts taking HP tablets and a common NSAID. There were no discernible differences in the pain scores, and the researchers did not compare the long-term gastrointestinal impacts among the two cohorts [361]. A larger, multicenter study conducted in Poland investigated the supplemental efficacy of a multicomponent gel containing HP extract with conventional OA therapy, with suitable pain scores and improved functional capacity [362]. Despite promising results, the long-term safety of HP extract supplements has not been determined and warrants further investigation [363].

### 5.5. Aescin

Like *Boswellia serrata* extract, aescin is also a terpenoid composed of two forms, α-escin and β-escin [364]. Aescin itself is extracted from the fruit and the seeds of *Aesculus hippocastanum*, commonly known as horse chestnut. Aescin has been shown to be effective against edema, inflammation, and various tumors [365,366,367,368]. Aescin exerts its anti-inflammatory role by promoting the uptake of the Glucocorticoid receptor through the cell membrane, which then inhibits the release of TNF-α, IL-1β, and IL-6 [369]. Much like HP extract, it can also act through the inhibition of NF-κB [370].

In a large prospective study conducted in Russia and Ukraine, researchers found that replacing the NSAID diclofenac with a topical cream formulation of aescin helped alleviate pain in OA patients of various clinical stages. However, this study did not have control groups, and the pain score questionnaires were obtained from the physicians rather than the patients themselves [371]. In a later trial, Zeng and colleagues investigated the impact of sodium aescinate, which is a commercial form of aescin, on OA patients while comparing it with conventional NSAID therapy. The researchers found that there was a statistically significant difference in pain alleviation and joint functioning in the intervention group as compared to the control [372]. A similar effect was observed earlier by Maghsoudi et al., albeit in an in vitro synoviocyte model [373].

While studies specifically directed against OA are sparse, the gastroprotective ability of aescin demonstrated in other cellular and patient-specific models is a lucrative prospect for its long-term use [374,375].

### 5.6. Matricaria chamomilla

*Matricaria chamomilla*, usually called chamomile, is a well-known medicinal plant of the Asteraceae family. Native to southern and eastern Europe and northern and western Asia, today it is widely distributed throughout the world [376,377]. The chemical compounds of this plant include apigenin, apigenin-7-O-glucoside (APG), caffeic acid, chlorogenic acid, luteolin, and luteolin-7-O-glucoside, terpene bisabolol farnesene, chamazulene, flavonoids (including apigenin, quercetin, patuletin, and luteolin), and coumarin [378,379,380,381,382].

Pharmacological investigations have reported several biological activities of *M. chamomilla*, and its possible use in various fields, including medicine, has been investigated. [383]. Several in vivo studies have demonstrated its therapeutic effect on a wide range of pathologies, including nervous diseases, diabetes, metabolic disorders, cardiovascular diseases, and allergies [384,385,386,387,388].

The plant has also been shown to relieve pain, heal wounds, and act as a protective agent for kidneys and liver, as well as gastrointestinal and reproductive systems [389,390,391]. Additionally, *M. chamomile* has anti-inflammatory and antioxidant activities and strong antiplatelet and anticarcinogenic properties. It can heal skin lesions and is beneficial for anxiety disorders [167,392,393,394,395,396,397].

In vitro, chamomile has been shown to inhibit free radical level formation following H_2_O_2_ treatments in human skin fibroblasts, as well as TNF-α production [398,399].

On the other hand, in vivo studies have also shown its capability to improve joint function and reduce knee and back pain [400]. The component that would appear to play a crucial role in chamomile’s anti-inflammatory effects is apigenin. Shoara et al. (2015) showed a significant beneficial effect of a traditional topical formulation of chamomile flower oil on analgesic use in patients with knee OA [401]. Additionally, chamomile oil showed some beneficial effects also on patients’ pain, stiffness, and physical activity. It has been observed that apigenin can have a strong inhibitory effect on prostaglandin E2 levels [402].

Finally, chamomile has been shown to interfere with the COX-2 pathway with a mechanism similar to those exerted by NSAIDs [403]. In addition to chamomile flavonoids and essential oils being able to penetrate the skin layers [404], chamomile has also shown anti-inflammatory and analgesic effects when applied topically [405].

### 5.7. Glycine soja

*Glycine soja* (GS), also known as wild soybean, is an annual climbing herb of the legume family (Fabaceae) [406]. It is native to East Asia, the Russian Far East, eastern China, the Korean peninsula, and Japan [407]. Considered the progenitor of cultivated soybean, GS has been used in China for more than 2000 years and is considered an excellent source of soy-derived drugs [408].

GS contains a wide range of compounds, including saponins and isoflavones (e.g., daidzein, 6-hydroxy-daidzein, daidzein glycosides, genistein, genistein glycosides, glycitein, and glycitein glycosides) [409]. Health benefits associated with soy polyphenols are attributed to phenolic acids, flavonoids, and anthocyanins [410]. Soy isoflavones are of particular interest in the pharmaceutical industry. GS shows various biologically relevant effects, improving blood lipid profile and reducing hepatic steatosis and adipocyte size in mice exposed to a high-fat diet [411].

Yun Mi Lee and collaborators studied both in vitro and in vivo the effects of GS leaves and stems (GSLSs) on OA, focusing on inflammation and ECM degradation [406]. In particular, the anti-inflammatory effects of GSLSs were investigated in SW1353 human chondrocytes stimulated with IL-1β, and significant reductions in the levels of the inflammatory mediators PGE2, IL-1β, IL-6, and TNF-α were shown following the treatments [412].

GSLSs also inhibit the expression of inflammatory cytokines and matrix metalloproteinases, with a protective role in collagen type II degradation, as observed in IL-1β-stimulated chondrocytes [406,413]. The chondro-protective role of GSLSs is due to the inhibition of NF-κB activation [414]. Collectively, GSLSs significantly reduce OA-associated joint pain, also lowering serum levels of proinflammatory mediators, cytokines, and matrix metalloproteinases. Thus, GSLSs represent a useful therapeutic candidate for OA.

### 5.8. Zingiber officinale Roscoe

*Zingiber officinale Roscoe*, commonly known as ginger, belongs to the *Zingiberaceae* family and has been used as a spice and herbal remedy for years. Its rhizome is a particularly rich source of bioactive substances used in Ayurvedic and Chinese medicine [415]. The pharmacological properties of ginger are related to numerous active phytocompounds belonging to the phenols and terpenes. The rhizomes of ginger plants comprise two different types of compounds. The first is the non-volatile oleoresin, the source of ginger’s pungent taste, and the second is the volatile essential oils [416]. Oleoresin comprises the main physiologically active substances of this spice, such as gingerols, shogaols, paradols, and zingerone [417]. One of the main components of ginger is 6-gingerol, which has anti-inflammatory and analgesic properties [418,419].

Preclinical studies have shown that the phytochemical compounds of ginger, including 6-shogaol, zingerone, and cedrol, are effective anti-rheumatic agents, as they alter signaling pathways involved in OA pathophysiology [420].

It has been shown that gingerols, shogaols, paradols, and other polyphenols present in ginger, together with sesquiterpenes, inhibit TNF-α, IL-1β, IL-2, IL-4, IL-6, IL-17, PGE 2, and COX-2 in human synoviocytes; this modulates the activation of NF-κB and the degradation of its inhibitor IkB-α [421,422,423,424,425,426]. Additionally, many studies highlight the antioxidant effects of ginger [427]. Its phenolic constituent, 6-gingerol, inhibited LPS-induced iNOS expression and the production of NO and other reactive nitrogen species in macrophages [428]. Therefore, gingerols and their derivatives may represent an alternative to NSAIDs without serious gastrointestinal or renal side effects. Naderi and coworkers concluded that ginger may be recommended as an appropriate supplement for patients with OA, as it is useful for reducing pain, stiffness, and inflammation in patients with OA [429].

### 5.9. Quercetin

Quercetin, a flavonoid widely distributed in fruits, herbs, and vegetables, has demonstrated a wide range of beneficial health properties. These include anti-inflammatory, immunomodulatory, and antioxidant effects, as well as contingent anti-arthritic and joint-protective properties in inflammatory joint diseases [300,430,431]. Recently, several studies have highlighted the therapeutic potential of quercetin in OA, effectively inhibiting inflammation and apoptosis of chondrocytes and thus preventing disease progression [432,433]. Quercetin has been identified as a potent senolytic drug capable of inducing apoptosis of senescent cells [434].

Understanding the molecular mechanisms involved in the action of quercetin is crucial to developing new targeted therapeutic strategies for the management of OA and maximizing its clinical potential in inflammatory joint diseases.

Quercetin has also been shown to inhibit endoplasmic reticulum stress-related cartilage degeneration, a key process in the pathogenesis of OA. This was demonstrated through the activation of the SIRT1/AMPK signaling pathway, which led to the prevention of OA progression in rat models [435]. Li and coworkers have also demonstrated in vivo the ability of quercetin to suppress the IRAK1/NLRP3 signaling pathway, reducing the levels of proinflammatory cytokines in OA [436]. Furthermore, quercetin demonstrates significant chondroprotective effects, reducing cartilage degradation and apoptosis of chondrocytes and promoting the synthesis of glycosaminoglycans to enhance the repair of damaged cartilage [432]. Wang and colleagues suggested that these effects are associated with the molecule’s ability to inhibit the p38 MAPK and ADAMTSs signaling pathway, thereby reducing relevant inflammatory factors, and promoting the expression of type II collagen to promote cartilage regeneration [437]. Finally, quercetin has been shown to inhibit MMP activity in OA [438].

## 6. Challenges in Natural Molecules Quality Control and Standardization

With the increasing demand for natural extracts and nutraceuticals for health-related applications, more attention is being paid to quality assurance and standardization of preparations. Despite the importance of the sector, also underlined by the World Health Organization’s Strategy on Traditional Medicine 2014–2023, discrepancies between regulatory bodies and manufacturers regarding the amount of quality testing required for dietary supplements represent a significant challenge in the industry [439]. Botanical extracts and mixtures pose unique challenges in detecting identification errors and contamination, both biological and chemical in nature. The lack of analytical methods and reference standards for the numerous bioactive ingredients present in dietary supplements is a significant scientific challenge. There is still no universal agreement on the acceptance of a single officially approved analysis method, as there are divergent opinions on who should be responsible for developing standards and analytical methods [440].

To ensure the safety of botanical dietary supplements, it is essential to implement stringent quality control and assurance measures throughout the entire production process. This includes sourcing botanical materials from reliable suppliers, with careful authentication of plant species through macroscopic and microscopic botanical examinations.

During the production of nutraceuticals, the use of solvents, additives, and purification techniques can be influenced by the presence of contaminants such as pesticides, herbicides, and heavy metals, which are known to cause serious adverse effects, including liver and kidney toxicity and even carcinogenicity [441,442]. Therefore, the safety of such products has become a priority for regulatory authorities. Although the use of pesticides is necessary to preserve the quality of medicinal herbs, it is crucial to adhere to the WHO guidelines regarding the presence of environmental contaminants in the final herbal products [443]. It is crucial to define and implement rigorous production procedures, ensuring standardization and quality control throughout all stages of the process [442].

After processing, botanical dietary supplements must be carefully analyzed to identify and remove any hazardous contaminants, such as pesticides, herbicides, and heavy metals, and undergo chromatographic tests to exclude the presence of unintended pharmaceutical contaminations. Chemical standardization, based on the concentration of active ingredients, and biological standardization, through in vitro and in vivo assays, are crucial to ensure the safety and reproducibility of botanical dietary supplements, providing consumers with products containing consistent levels of active ingredients and predictable pharmacological effects [440]. However, the uniform and coherent regulation of such products still poses a challenge, as global standards governing their production, sale, and marketing are lacking, highlighting the need for international consensus on how to define and regulate this category of products [444,445].

## 7. Future Research Directions

The beneficial effects of natural molecules are numerous, offering promising options for complementing established treatment approaches. As widely discussed in the former section of the article, many of these molecules show beneficial effects in addressing OA, targeting pathways implicated in joint degradation and underlying pathological mechanisms. Inflammation and oxidative stress, recognized as paramount elements in OA contexts, represent an important target to slow tissue degeneration and improve patients’ QoL [446]. The listed natural extracts, which represent only the most important among an incredibly wide range of molecules used in traditional medicine to counteract OA, converge to alleviate these pathological elements through pleiotropic mechanisms [447,448]. Their integration in clinical and classical therapeutical regimes could therefore reduce the dosage and frequency of use of molecules such as NSAIDs, as well as their AEs.

Regarding natural molecules, it is critical to recognize that merely identifying those with significant beneficial effects for a particular disorder is not sufficient. Equally important is the evaluation of optimal dosage, formulation, and absorption efficiency in the body [449]. This factor could partially account for the significant variability observed in clinical trial outcomes or, in some cases, the absence of conclusive results altogether. The lack of objective methods to accurately determine disease status and measure treatment-related improvements, excluding patients’ reported outcomes, contributes to the problem. Two further elements could provide a strong improvement in OA management and treatment. A better comprehension of OA early-stage processes, considering the heterogeneity of its manifestations and progression in the population, is required. It is also important to abandon “one-size-fits-all” therapeutical approaches to others that are more tailored to patients.

Tailoring therapies to patients may mean modulating the number of drugs to be used and the number of daily administrations. Reaching the innermost layers of the joint may present difficulties, however [450]. So, the use of a targeted and slow-release pharmacological approach may be considered. Formulations such as microemulsions, liposomes, sequessomes, solid lipid nanoparticles, or nanostructured lipid carriers have been studied in recent years with great interest. To date, however, only one phase II clinical trial based on the use of diclofenac in the form of nanoemulsion cream results [451].

Similarly promising but extremely cutting-edge could be the use of plant extracellular vesicles (PEVs) in the therapy of OA. In contrast to their animal counterparts, mammalian cell-derived extracellular vesicles (MEVs), and synthetic carriers, PEVs have the advantage of being non-immunogenic. These molecules can be isolated through numerous methods from a variety of edible plants, retaining many molecules with potent biological activities such as nucleic acids, lipids, and metabolites within them [452]. The anti-inflammatory and immunomodulatory potential demonstrated is certainly attractive and is suitable for use in OA therapy. However, it will still take a long time before safety studies open them up for human use and for the technology to overcome the many bioproduction and plant bioengineering challenges that currently limit large-scale production.

## 8. Conclusions

OA is a progressive condition associated with the destruction of the joint cartilage, and no resolutive therapies are clinically available nowadays. The main problem, however, is represented by the disability following the joint loss of function and the increasing and constant pain deriving from the process. The use of painkillers has a limited and temporary effect, acting just on OA symptoms and not on the causes generating the suffering. In the last decades, several approaches have been developed to reduce the degeneration process of the cartilage as well as the pain by acting directly inside the joint when directly injected into it. Also, in this case, the benefits appear limited, and clinical trials have not been able to prove their efficacy and safety up until now. In such a context, several natural molecules have been used for a very long time for their beneficial properties as anti-inflammatories and antioxidants.

OA is a progressive condition associated with several causative factors, destroying joint cartilage. Therapeutic strategies are directed against the palliative management of the disease alone, and no definitive cure exists. As the disability followed by the joint loss of function leads to severe pain, painkillers are routinely prescribed and have a limited and temporary impact while causing several side effects. Moreover, several other approaches have also been adapted, for instance, the injection of strengthening factors like HA or PRP, with limited clinical success. Natural plant extracts and nutraceuticals have been hailed as potent alternative therapies for OA because of their intrinsic anti-inflammatory and antioxidant properties. Recent evidence has shown them to be effective at alleviating pain through the inhibition of proinflammatory pathways like the NF-κB one. Moreover, they also offer a direct effect in promoting cartilage protection by mitigating MMP activity and promotion mechanisms of autophagy, favoring the removal of damaged chondrons. At the state of the art, more focused studies and trials are, however, required to consolidate evidence in their favor and in perspective for recommending clinical guidelines for their use.

## Figures and Tables

**Table 1:** Kellgren and Lawrence classification of radiological OA. The pathology is present starting from grade 2, even if of minimal severity [71].

Grade	KL Scale
0 None	No pathophysiologic involvement of osteoarthritis
1 Doubtful	Normal joint with only one tiny osteophyte
2 Minimal	Clear osteophytes at two spots with slight hardening of the bone under the cartilage and possible hollow areas, but normal joint gap and no distortion
3 Moderate	Moderate osteophytes presence, some bone malformation and shrinking of joint gap
4 Severe	Large presence of osteophytes and bone end impairment, loss of joint space, densification, and cysts

**Table 2:** Clinical trials on natural molecules in OA (information extracted from clinicaltrials.gov, accessed on 31 March 2024).

Molecule Class	Mechanisms of Action	Clinical Studies	ClinicalTrials.gov Identifier	Status	Outcome	Sponsor/ Collaborators
*Curcumin*						
Diarylheptanoid	Anti-inflammatory effect [243]	The Efficacy and Safety of Curcuma Domestica Extracts and Ibuprofen for Therapy of Patients with Knee Osteoarthritis	NCT00792818	Phase 3—Completed N = 367	Pain reduction and functional improvement comparable to ibuprofen but with lesser gastrointestinal side effects [297]	Mahidol University, Salaya, Thailand
		Evaluation of FLEXOFYTOL^®^ Versus PLACEBO (COPRA)	NCT02909621	Phase 2—Completed N = 150	Pain reduction when used as adjuvant to paracetamol and/or NSAIDs in comparison to placebo; suitable safety profile and lower Patient Global Assessment of Disease Activity reported in comparison to placebo [298]	Tilman S.A., Baillonville, Belgium
		Comparative Study of Turmeric Extract in Patients with Arthrosis	NCT04500210	Phase 3—Completed N = 120	-	Kaj Winther Hansen
		Evaluation of the Efficacy of a Turmeric Extract (Arantal^®^) in Patients with Osteoarthritis of the Knee (Gonarthrosis)	NCT00992004	Phase 2—Completed N = 280	-	Bioxtract S.A., Gembloux, Belgium
		Exploratory Non-Comparative Study to Evaluate the Efficacy of Highly Bioavailable Curcumin (Flexofytol) in Patients with Knee Osteoarthritis	NCT01909037	Early Phase 1—Completed N = 22	Chondrogenic effect and inhibition of proinflammatory cytokines, prostanoids, and MMPs released by chondrocytes. Inhibition of TNF-α activity and production both in vitro and in vivo [299]	Tilman S.A., Baillonville, Belgium
		Effectiveness of Curcumin-based Food Supplement in Reducing Pain and Inflammatory Component in Osteoarthritis (FENOXI-1900)	NCT04207021	Not Applicable N = 134	-	KOS Care SRL—Istituto di Riabilitazione Santo Stefano
		Combination of Curcuminoid with Acupressure for Inflammation and Pain in the Elderly with Osteoarthritis Genu	NCT06105840	Phase 2—Enrolling by invitation N = 70	-	Gadjah Mada University, Sleman, Indonesia
		Randomized Trial of Regenexx Stem Cell Support Formula	NCT04661267	Not Applicable N = 80	-	Regenexx LLC, Des Moines, IA, USA
		Epigenorm Antivir Combined with Acupuncture for the Treatment of Osteoarthritis Patients Who Are Overweight or Obese	NCT03540186	Not Applicable N = 15	-	Epigenorm Antivir Combined with Acupuncture for the Treatment of Osteoarthritis Patients Who Are Overweight or Obese
*Bromelain*						
Proteinase-peptidase	Anti-inflammatory, analgesic, anti-edematous, and fibrinolytic effects [244]	Study to Investigate the Mechanism of Action of an Oral Enzyme Treatment with Bromelain, Trypsin and Rutoside Versus Placebo in Subjects with OsTeoarthritis (WobeSmart)	NCT05038410	Not Applicable N = 40	-	Mucos Pharma GmbH & Co. KG, Berlin, Germany
*Harpagophytum procumbens (devil’s claw)*						
Mix of phenolic acids and glycosides, triterpenes, phytosterols, iridoid glucosides like harpagoside and various flavonoids	Anti-rheumatic, anti-inflammatory, and analgesic effects [245]	Trial Evaluating Devil’s Claw for the Treatment of Hip and Knee Osteoarthritis	NCT00295490	Phase 2—Completed N = 67	-	University of Southampton, UK
		Clinical Efficacy and Safety of Loxacon Dietary Supplement Capsules at Patients with Knee Arthrosis	NCT05925725	Phase 4—Completed N = 100	-	Polyclinic of the Hospitaller Brothers of St. John of God, Budapest, Hungary
*Boswellia serrata*						
Terpene	Anti-inflammatory effect [246]	Efficacy of Myalgesin™ to Support Joint Function in Patients with Knee Osteoarthritis	NCT00577330	Phase 3—Not applicable N = 110	-	ProThera, Inc., Reno, NV, USA
		A Study of the Feasibility of Using the Dietary Supplement “ARTNEO” in Patients with Osteoathritis	NCT05975879	Not Applicable N = 212	-	NPO Petrovax, Moscow, Russia
		Clinical Efficacy and Safety of Loxacon Dietary Supplement Capsules at Patients with Knee Arthrosis	NCT05925725	Phase 4—Completed N = 100	-	Polyclinic of the Hospitaller Brothers of St. John of God, Budapest, Hungary
		A Study to Assess Efficacy of Supporting Properties and Safety of ARTNEO in Patients with Knee Osteoarthritis	NCT06032442	Not Applicable N = 70	-	NPO Petrovax, Moscow, Russia
		Management of Joint Pain Associated with Osteoarthritis of the Knee with Association of Plant Extracts	NCT02977936	Not Applicable N = 126	-	PiLeJe, Paris, France
		To Assess the Lanconone^®^ (E-OA-07) Efficacy in Physical Activity-related Pain-LEAP Study (LEAP)	NCT03262805	Not Applicable N = 73	-	Vedic Lifesciences Pvt. Ltd., Mumbai, India
		Effects of Glucosamine and Chondroitin Supplementation in Women with Knee Osteoarthritis Participating in an Exercise and Weight Loss Program	NCT01271218	Phase 4—Completed N = 36	-	Texas A&M University, College Station, TX, USA
*Quercetin*						
Flavonoid	Antioxidant [300]	Effect of Natural Senolytic Agents & NLRP3 Inhibitors on Osteoarthritis	NCT05276895	Not Applicable N = 60	Ongoing	Assiut University, Assiut, Egypt

## Data Availability

No new data were created or analyzed in this study. Data sharing is not applicable to this article.
